# Characterization of Avian Influenza Viruses A (H5N1) from Wild Birds, Hong Kong, 2004–2008

**DOI:** 10.3201/eid1503.081190

**Published:** 2009-03

**Authors:** Gavin J.D. Smith, Dhanasekaran Vijaykrishna, Trevor M. Ellis, Kitman C. Dyrting, Y.H. Connie Leung, Justin Bahl, Chun W. Wong, Huang Kai, Mary K.W. Chow, Lian Duan, Allen S.L. Chan, Li Juan Zhang, Honglin Chen, Geraldine S.M. Luk, J.S. Malik Peiris, Yi Guan

**Affiliations:** State Key Laboratory of Emerging Infectious Diseases/The University of Hong Kong, Hong Kong Special Administrative Region, People’s Republic of China (G.J.D. Smith, D. Vijaykrishna, Y.H.C. Leung, J. Bahl, H. Kai, L. Duan, L.J. Zhang, H. Chen, J.S.M. Peiris, Y. Guan); Agriculture, Fisheries and Conservation Department, Hong Kong (T.M. Ellis, K.C. Dyrting, C.W. Wong, M.K.W. Chow, A.S.L. Chan, G.S.M. Luk); HKU-Pasteur Research Centre, Pokfulam, Hong Kong (J.S. Malik Peiris); 1 These authors contributed equally to this article.

**Keywords:** Highly pathogenic avian influenza, virus evolution, molecular epidemiology, Hong Kong, research

## Abstract

From January 2004 through June 2008, surveillance of dead wild birds in Hong Kong, People’s Republic of China, periodically detected highly pathogenic avian influenza (HPAI) viruses (H5N1) in individual birds from different species. During this period, no viruses of subtype H5N1 were detected in poultry on farms and in markets in Hong Kong despite intensive surveillance. Thus, these findings in wild birds demonstrate the potential for wild birds to disseminate HPAI viruses (H5N1) to areas otherwise free from the viruses. Genetic and antigenic characterization of 47 HPAI (H5N1) viruses isolated from dead wild birds in Hong Kong showed that these isolates belonged to 2 antigenically distinct virus groups: clades 2.3.4 and 2.3.2. Although research has shown that clade 2.3.4 viruses are established in poultry in Asia, the emergence of clade 2.3.2 viruses in nonpasserine birds from Hong Kong, Japan, and Russia raises the possibility that this virus lineage may have become established in wild birds.

## Abstract 2

Repeated detection of subclade 2.3.2 viruses in nonpasserine birds from different regions suggests possible establishment of this lineage in wild bird species.

Highly pathogenic avian influenza (HPAI) viruses (H5N1) derived from the goose/Guangdong/1/96 (Gs/GD) lineage have spread to more than 60 countries across Eurasia and Africa (*1*–*3*). The unprecedented panzootic caused by the HPAI viruses (H5N1) has been mediated by the movement of poultry and poultry products and, in some instances (e.g., clade 2.2 viruses), by wild bird migration (*4*–*6*). After introduction, the viruses became endemic in some countries, causing repeated poultry outbreaks and spilling over to cause zoonotic infection in humans, thus posing a persistent potential pandemic threat (*7*–*9*). However, in some affected countries with substantial resources (e.g., Japan and South Korea), despite the repeated introduction of subtype H5N1 viruses that have occasionally led to associated outbreaks in poultry, early and aggressive intervention measures prevented these viruses from becoming endemic in poultry, and no human cases were detected (*2*,*10*–*13*).

HPAI viruses (H5N1) were first observed to cause outbreaks of disease in wild and captive birds in Penfold and Kowloon Parks, Hong Kong, in late 2002 and in 2003 (*14*). The Kowloon Park outbreak was concurrent with outbreaks caused by this virus in several live poultry markets and on some chicken farms in Hong Kong (*14*). Measures to improve biosecurity on farms, changes in the poultry marketing system, the introduction of rest days in poultry markets, and vaccination for all poultry entering Hong Kong markets have prevented subsequent HPAI (H5N1) outbreaks in farmed poultry in Hong Kong (*15*). No further cases of infection in live poultry markets were detected from November 2003 through June 2008, when live bird market surveillance detected incursion of a new HPAI (H5N1) virus (*2*).

The avian influenza control program in Hong Kong includes intensive active surveillance of live poultry markets, aviary bird markets, poultry farms, and migratory birds at several wetland sites in Hong Kong (*16*). In addition, avian influenza surveillance has been conducted on wild birds found dead (wild birds and caged birds released for ceremonial purposes are collectively referred to as wild birds in this article) (*17*). Until the recent incursion of HPAI virus (H5N1) in June 2008, no viruses of subtype H5N1 had been detected on poultry farms or in markets in Hong Kong since November 2003, although 2 HPAI viruses (H5N1) were detected in chickens smuggled into Hong Kong in 2006 (*18*). However, HPAI viruses (H5N1) have been detected every year in a variety of dead wild birds, including falcons, egrets, herons, and various passerine species (*1*,*4*,*7*,*14*,*18*,*19*).

In this study, we antigenically and genetically characterized all HPAI (H5N1) viruses isolated from the dead bird surveillance program in Hong Kong to gain insights into the evolutionary history and possible transmission pathways of the viruses. Our research shows that viruses isolated each winter from 2004 through 2007 were genetically distinct, belonging to different subtype H5N1 clades. These different clades suggest multiple introductions of HPAI virus (H5N1) reassortments into Hong Kong through wild birds. This study also demonstrates that wild birds can disseminate the HPAI virus (H5N1) and have the potential to seed areas otherwise free from the virus.

## Materials and Methods

### Virus Isolation and Characterization

Viruses were isolated from specimens obtained from dead wild birds (Appendix Table) by inoculating embryonated eggs at the laboratory of the Agriculture, Fisheries and Conservation Department of the Hong Kong SAR Government. Viruses were identified by real time–PCR and by standard hemagglutination-inhibition (HI) tests using a panel of World Organization for Animal Health’s Avian Influenza Reference Laboratory antisera (Veterinary Laboratory Agency, Weybridge, UK) as previously described (*14*,*23*,*24*). All virus isolation was conducted in biosafety level 3 facilities. Details of the avian influenza (H5N1) surveillance program in Hong Kong for dead wild birds, including pathologic findings and diagnostic testing, are reported separately (*17*).

### Antigenic Analysis

Antigenic characterization of the influenza viruses (H5N1) was carried out by HI assay using 5 ferret polyclonal antisera, as previously described (*24*). The ferret antisera were provided by St Jude Children’s Research Hospital (Memphis, TN, USA) (duck/Hunan/101/2004 and muscovy duck/Vietnam/1455/2006) and by the Centers for Disease Control and Prevention (Atlanta, GA, USA) (Anhui/1/2005, Indonesia/5/2005, Indonesia/CDC357/2006, Vietnam/1203/2004, and whooper swan/Mongolia/244/2005). The HI assay started at a serum dilution of 1:40.

### Phylogenetic and Molecular Analysis

To understand the evolutionary history of avian influenza viruses (H5N1) isolated from wild birds in Hong Kong, we conducted whole genome sequencing of 29 avian influenza viruses (H5N1) that were isolated from dead wild birds in 2006–2008. All 8 gene segments of these viruses were characterized and phylogenetically analyzed. These data were compared with the virus sequence data for an additional 18 influenza viruses (H5N1) isolated from dead wild birds in Hong Kong in 2004–2008, with virus sequence data for the 2 viruses obtained from chickens smuggled into Hong Kong in 2006, and with all other available sequence data from the NCBI Influenza Virus Resource (*25*).

Viral RNA extraction, cDNA synthesis, PCR, and sequencing were carried out as described previously (*19*). Sequences were assembled and edited with Lasergene version 7.2 (DNASTAR, Madison, WI, USA). Se-Al version 2.0 was used for alignment and residue analysis (http://tree.bio.ed.ac.uk/software/seal). The program MrModeltest version 2.2 was used to determine the appropriate DNA substitution model and rate heterogeneity (*26*). The generated model was used in all subsequent analyses. Neighbor-joining trees were constructed with PAUP* version 4.0b (*27*), and Bayesian analysis was conducted with MrBayes version 3.1.2 (*28*) by using 2 replicates of 1 million generations with 6 chains, sampling every 100 generations. The convergence of chains and the estimation of burn-in were assessed using Tracer version 1.4 (http://beast.bio.ed.ac.uk). Estimates of the phylogenies were calculated by performing 1,000 neighbor-joining bootstrap replicates, and Bayesian posterior probabilities were calculated from the consensus of 18,000 trees after excluding the first 2,000 trees as burn-in. The full-genome sequences of 29 influenza viruses (H5N1) obtained in this study are available from GenBank under accession nos. CY036042–CY036273.

## Results

### Virus Isolation

From early 2004 through June 2008, most isolates of influenza virus (H5N1) from dead wild birds were detected during the cooler months (i.e., from December to the following February) (Appendix Table). Almost all positive samples of influenza virus subtype H5N1 were isolated from a variety of dead wild birds, including falcons, egrets, herons, and various passerine species. On 2 occasions, influenza virus (H5N1) was isolated from smuggled chickens (Appendix Table).

### Phylogenetic Analysis

To understand the molecular epidemiology of the viruses isolated from the dead birds, we conducted phylogenetic analysis of the hemagglutinin (HA), neuramindase (NA), and each of the 6 internal gene segments of the viruses, along with the Gs/GD-like HPAI viruses (H5N1) isolated from different regions of Hong Kong. In the HA gene tree, the wild bird viruses fell into 2 main groups, either clade 2.3.2 or 2.3.4, with the exception of 1 virus in clade 9 (Figure). The phylogenetic placement of these viruses corresponds well with the known evolution of the influenza virus subtype H5N1 that has been documented in Asia.

**Figure:**
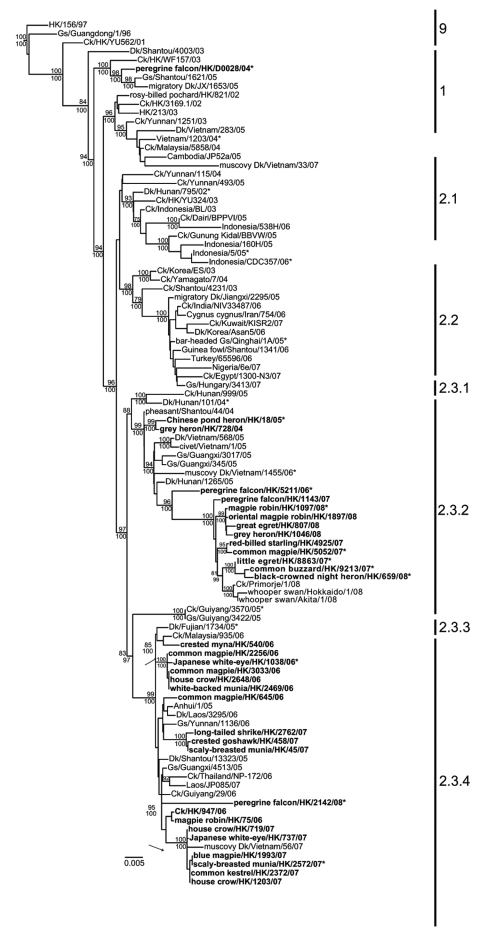
Phylogenetic relationships of the hemagglutinin genes of representative influenza viruses. Numbers above and below the branch nodes indicate neighbor-joining bootstrap values >70% and Bayesian posterior probabilities >95%, respectively. Not all supports are shown due to space constraints. Analyses were based on nt 49–1,677 and the tree rooted to duck/Hokkaido/51/1996. Numbers to the right of the figure refer to World Health Organization influenza (H5N1) clade designations (Appendix Table). Viruses isolated from wild birds and chickens in Hong Kong from 2004–2008 are in boldface. *Indicates viruses included in the antigenic analysis (Table). Scale bar indicates 0.01 nucleotide substitutions per site. Ck, chicken; Dk, duck; Gs, goose; HK, Hong Kong.

The isolate detected in early 2004 (peregrine falcon/HK/D0028/2004) clustered into clade 9, which includes viruses isolated from both poultry and migratory ducks during 2003–2005 in China (Figure). The 3 viruses in clade 2.3.2 (grey heron/HK/728/2004, grey heron/HK/837/2004, Chinese pond heron/HK/18/2005) that were isolated in late 2004/early 2005 were most closely related to viruses detected in southern China and Vietnam during the same period. However, 29 of 31 viruses isolated in early 2006 and early 2007 were closely related to clade 2.3.4 viruses (represented by Dk/Fujian/1734/2005), corresponding with the time of emergence and predominance of this virus lineage (Figure, Appendix Table). The wild bird viruses isolated from May 2007 through March 2008 belonged exclusively to clade 2.3.2, with the exception of the clade 2.3.4 virus peregrine falcon/HK/2142/2008. These clade 2.3.2 viruses (H5N1) were most closely related to previous isolates from dead wild birds from Hong Kong (peregrine falcon/HK/5211/2006 and peregrine falcon/HK/1143/2007) and to isolates from Japan and Russia (whooper swan/Hokkaido/1/2008, whooper swan/Akita/1/2008 and Ck/Primorje/1/2008) (Figure).

Phylogenetic analysis of the NA of these isolates showed a similar phylogenetic relationship to that observed for the HA (data not shown). These findings show that influenza viruses (H5N1) detected each winter from 2004 through 2007 were genetically distinct and belonged to different sublineages or clades, suggesting that multiple introductions occurred during the past 4 years.

Phylogenetic analyses of the internal gene complex showed that the viruses from dead wild birds in Hong Kong belonged to different subtype H5N1 genotypes (Appendix Table). The virus peregrine falcon/HK/D0028/2004 clustered with those genotype Z viruses isolated from poultry in mainland China during the same period, and the 3 viruses in clade 2.3.2 that were isolated in late 2004 and early 2005 belonged to genotype V2 (*22*). The 17 viruses in clade 2.3.4 that were detected in early 2006 belonged to either genotype V (n = 6) or G (n = 10). The viruses isolated from January through June 2007, both clades 2.3.2 and 2.3.4, were mostly genotype V (n = 15), although 3 genotype V1 viruses were also detected (Appendix Table). Genotypes V1 and V2 are reassortments of genotype V that have incorporated novel PB2 and PB1 genes (*22*). Eight viruses isolated from November 2007 through March 2008 also belonged to genotype V, and 2 isolates (little egret/HK/8550/2007 and peregrine falcon/HK/2142/2008) were novel reassortments. The genetic diversity of these viruses confirms the multiple introductions of influenza viruses (H5N1) to Hong Kong.

Two thirds (12/18) of the clade 2.3.2 viruses were isolated from nonpasserine hosts, mostly species of egret, heron, and raptors (Appendix Table). In contrast, 3 (11%) of 28 viruses in clade 2.3.4 were isolated from nonpasserine hosts, excluding the viruses from the 2 chickens. Although inconclusive, this pattern suggests that clade 2.3.2 viruses may have an adaptation that enables them to infect and cause disease in nonpasserine species more easily than in other bird species.

### Molecular Characterization

All 29 viruses characterized were highly pathogenic with variations of the multibasic cleavage site in the HA molecule. However, all clade 2.3.4 viruses had a Gln→Leu substitution at position –9 from the cleavage site (LRERRRK-RG), a factor consistent with previous reports (*18*). The receptor-binding pocket of the HA1 retains amino acid residues 222-Gln and 224-Gly (H5 numbering used throughout) that preferentially bind to α-2,3-NeuAcGal receptors (*29*–*31*). Other amino acid residues relevant to receptor-binding sites were identical to those of HK/156/1997 and Gs/GD-like viruses in most isolates. However, all clade 2.3.2 viruses characterized had an HA Ser129Leu substitution, a factor previously observed in both clade 1 and 2 viruses (*8*,*32*). The clade 2.3.2 virus grey heron/HK/3088/2007 also had a Lys212Arg substitution (*30*).

In the NA amino acid sequences, all isolates characterized had 274-His, indicating sensitivity to oseltamivir (*33*). One virus (common buzzard/HK/9213/2007) had a Ser31Asn substitution in the M2 protein, a change that may confer resistance to the adamantanes and that has been present in all Clade 1 viruses characterized to date. This substitution has also been sporadically detected in other H5N1 lineages (*34*). No amantadine-resistant mutations were observed in the remaining isolates. None of these viruses have the Lys627 residue commonly found in Qinghai-like (clade 2.2) viruses (*6*).

### Antigenic Analysis

Two of the clade 2.3.4 representative viruses (Japanese white-eye/HK/1038/2006 and scaly-breasted munia/HK/2572/2007) showed good reactivity against the clade 2.3.4 antiserum, but peregrine falcon/HK/2142/2008 was markedly less reactive with a >4-fold reduction in titer (Table). Also, peregrine falcon/HK/2142/2008 was poorly reactive against all ferret antisera tested. The pattern of reactivity of the clade 2.3.2 viruses from dead wild birds was similar to that of the homologous virus (muscovy duck/Vietnam/1455/2006) (Table).

**Table:** Antigenic analysis of influenza viruses A (H5N1) by hemagglutinin inhibition test, Hong Kong, China, 2008*

Virus	Clade†	Ferret antisera titers to:						
		VNM1203‡ (clade 1)	IDN5 (clade 2.1)	CDC357 (clade 2.1)	MNG244 (clade 2.2)	HN101 (clade 2.3.1)	VNM1455 (clade 2.3.2)	Anhui1 (clade 2.3.4)
VNM1203	1	**640**	40	<40	<40	80	40	80
Dk/Hunan/795/2002	2.1	80	640	320	160	160	160	<40
IDN5	2.1	40	**1,280**	640	80	40	160	160
CDC357	2.1	80	2,560	**1,280**	160	80	320	320
BHG/Qinghai/1A/2005	2.2	40	320	160	**320**	160	80	40
HN101	2.3.1	40	640	160	640	**640**	320	80
CPH/HK/18/2005	2.3.2	<40	40	<40	40	80	<40	<40
VNM1455	2.3.2	40	160	160	160	160	**320**	<40
Pfalcon/HK/5211/2006	2.3.2	<40	320	160	40	80	160	<40
Common magpie/HK/5052/2007	2.3.2	<40	320	160	160	80	320	<40
Common buzzard/HK/9213/2007	2.3.2	<40	160	80	80	80	160	<40
Little egret/HK/8863/2007	2.3.2	<40	160	80	160	80	320	<40
BCN heron/HK/659/2008	2.3.2	<40	160	80	80	80	320	<40
Magpie robin/HK/109720/08	2.3.2	<40	320	160	320	160	640	<40
Ck/Guiyang/3570/2005	2.3.3	160	160	40	160	640	160	640
Dk/Fujian/1734/2005	2.3.4	80	160	80	<40	80	40	**640**
JWE/HK/1038/2006	2.3.4	80	320	160	40	640	320	1,280
SB munia/HK/2572/2007	2.3.4	80	80	40	<40	80	<40	640
Pfalcon/HK/2142/2008	2.3.4	80	<40	<40	<40	<40	<40	40
Pfalcon/HK/D0028/2004	9	320	80	40	<40	80	<40	160

*VNM1203, Vietnam/1203/2004; IDN5, Indonesia/5/2005; CDC357, Indonesia/CDC357/2006; MNG244, whooper swan/Mongolia/244/2005; HN101, duck/Hunan/101/2004; VNM1455, muscovy duck/Vietnam/1455/2006; Anhui1, Anhui/1/2005; Dk, duck; BHG, bar-headed goose; CPH, Chinese pond heron; HK, Hong Kong; Pfalcon, peregrine falcon; BCN heron, black-crowned night heron; Ck, chicken; JWE, Japanese white-eye; SB munia, scaly-breasted munia. **Boldface** numbers indicate titers to prototype viruses. †Clade designations according to the World Health Organization influenza (H5N1) nomenclature system (*21*). ‡Ferret antisera dilution started at 1:40.

## Discussion

Genetic and antigenic characterization of HPAI wild bird viruses (H5N1) suggests that they are closely related to viruses isolated in Asia during the same time (*1*,*7*,*18*). During this period, an intensive avian influenza (H5N1) surveillance program was conducted concurrently on poultry farms and at markets in Hong Kong, and no subtype H5N1 viruses were detected from late 2003 until June 2008, when it was detected in fecal droppings in retail poultry markets (*2*). Thus, the repeated finding of influenza virus (H5N1) from dead wild birds in the absence of local poultry infection demonstrates the potential of wild birds to disperse the virus over at least moderate distances (i.e., tens or hundreds of kilometers).

The present study also demonstrates the role of the Hong Kong SAR as a sentinel for detecting emerging infectious diseases in Asia. It further demonstrates that surveillance of avian influenza virus (H5N1) in dead wild birds can play a key role as an early warning system for the introduction of this virus, a factor consistent with experience elsewhere (e.g., in Germany, United Kingdom, Russia). A similar strategy of conducting surveillance on wild birds would be useful for other regions in monitoring for these viruses that have the potential to infect a wide range of hosts, including humans (*2*,*35*).

Viruses isolated from January through March 2007 were, with 1 exception, clade 2.3.4 viruses, and were mostly isolated from passerine birds. From 2005 through 2007, clade 2.3.4 viruses became the dominant virus detected in live poultry markets in southern China and were detected in outbreaks of disease in poultry in Laos, Malaysia, Thailand, and northern Vietnam (*2*,*19*,*36*). However, the emergence of clade 2.3.2 viruses as the only viruses detected in wild birds, both passerine and nonpasserine, in the winter of 2007/2008 in Hong Kong is notable. Whether the detection of this clade reflects a dominance of this virus within poultry flocks in the wider region is unknown because little recent genetic data on influenza viruses (H5N1) are available from poultry in the region. Alternatively, the clade 2.3.2 is possibly adapted to wild birds, just as the clade 2.2 viruses appear to be (*37*). Phylogenetically similar clade 2.3.2 viruses of subtype H5N1 have been recently isolated from dead wild swans (whooper swan/Hokkaido/1/2008 and whooper swan/Akita/1/2008) in Japan and from chicken in Russia (Ck/Primorje/1/2008).

The establishment of another influenza virus (H5N1) lineage in wild birds, if indeed this establishment has occurred, has potentially far reaching consequences with the possibility of the long range spread of clade 2.3.2 viruses in a manner similar to the spread of clade 2.2 viruses (*2*,*6*,*37*). This potential for spread, along with the fact that some clade 2.3.2 viruses are antigenically distant from current avian influenza vaccine candidates, highlights why a clade 2.3.2 virus, common magpie/HK/5052/2007, has been recently recommended as an avian influenza (H5N1) vaccine candidate by the World Health Organization (*38*). These developments indicate a need for more intensive surveillance in the region and may also have implications for vaccination programs for poultry.

## Supplementary Material

Appendix Table Influenza viruses A (H5N1) isolated in Hong Kong, China, 2004-2008

## References

[1] Li KS, Guan Y, Wang J, Smith GJ, Xu KM, Duan L, Genesis of a highly pathogenic and potentially pandemic H5N1 influenza virus in eastern Asia. Nature. 2004;430:209–13. 10.1038/nature02746 15241415

[2] Food and Agricultural Organization of the United Nations. FOAAIDEnews. Avian influenza disease emergency. Situation update 55. 2008 [cited 2008 Aug 28]. Available from http://www.fao.org/docs/eims/upload//246457/aj097e00.pdf

[3] World Health Organization Global Influenza Program Surveillance Network. Evolution of H5N1 avian influenza viruses in Asia. Emerg Infect Dis. 2005;11:1515–21. 16318689 10.3201/eid1110.050644 PMC3366754

[4] Guan Y, Peiris JS, Lipatov AS, Ellis TM, Dyrting KC, Krauss S, Emergence of multiple genotypes of H5N1 avian influenza viruses in Hong Kong SAR. Proc Natl Acad Sci U S A. 2002;99:8950–5. 10.1073/pnas.132268999 12077307 PMC124404

[5] Tumpey TM, Suarez DL, Perkins LEL, Senne DA, Lee JG, Lee YJ, Characterization of a highly pathogenic H5N1 avian influenza A virus isolated from duck meat. J Virol. 2002;76:6344–55. 10.1128/JVI.76.12.6344-6355.2002 12021367 PMC136198

[6] Chen H, Smith GJD, Zhang SY, Qin K, Wang J, Li KS, H5N1 virus outbreak in migratory waterfowl. Nature. 2005;436:191–2. 10.1038/nature03974 16007072

[7] Chen H, Smith GJ, Li KS, Wang J, Fan XF, Rayner JM, Establishment of multiple sub-lineages of H5N1 influenza virus in Asia—implications for pandemic control. Proc Natl Acad Sci U S A. 2006;103:2845–50. 10.1073/pnas.0511120103 16473931 PMC1413830

[8] Smith GJ, Naipospos TS, Nguyen TD, de Jong MD, Vijaykrishna D, Usman TB, Evolution and adaptation of H5N1 influenza virus in avian and human hosts in Indonesia and Vietnam. Virology. 2006;350:258–68. 10.1016/j.virol.2006.03.048 16713612

[9] Tran TH, Nguyen TL, Nguyen TD, Luong TS, Pham PM, Nguyen VC, Avian influenza A (H5N1) in 10 patients in Vietnam. N Engl J Med. 2004;350:1179–88. 10.1056/NEJMoa040419 14985470

[10] Lee CW, Suarez DL, Tumpey TM, Sung HW, Kwon YK, Lee YJ, Characterization of highly pathogenic H5N1 avian influenza A viruses isolated from South Korea. J Virol. 2005;79:3692–702. 10.1128/JVI.79.6.3692-3702.2005 15731263 PMC1075707

[11] Mase M, Tsukamoto K, Imada T, Imai K, Tanimura N, Nakamura K, Characterization of H5N1 influenza A viruses isolated during the 2003–2004 influenza outbreaks in Japan. Virology. 2005;332:167–76. 10.1016/j.virol.2004.11.016 15661149

[12] Lee YJ, Choi YK, Kim YJ, Song MS, Jeong OM, Lee EK, Highly pathogenic avian influenza virus (H5N1) in domestic poultry and relationship with migratory birds, South Korea. Emerg Infect Dis. 2008;14:487–90. 10.3201/eid1403.070767 18325269 PMC2570817

[13] Sims LD. Experience in control of avian influenza in Asia. Dev Biol (Basel). 2007;130:39–43. 18411934

[14] Ellis TM, Bousfield RB, Bissett LA, Dyrting KC, Luk GSM, Tsim ST, Investigation of outbreaks of highly pathogenic H5N1 avian influenza in waterfowl and wild birds in Hong Kong in late 2002. Avian Pathol. 2004;33:492–505. 10.1080/03079450400003601 15545029

[15] Sims LD, Ellis TM, Liu KK, Dyrting K, Wong H, Peiris M, Avian influenza in Hong Kong 1997–2002. Avian Dis. 2003;47(Suppl):832–8. 10.1637/0005-2086-47.s3.832 14575073

[16] Sims LD, Guan Y, Ellis TM, Liu KK, Dyrting K, Wong H, An update on avian influenza in Hong Kong 2002. Avian Dis. 2003;47(Suppl):1083–6. 10.1637/0005-2086-47.s3.1083 14575116

[17] Ellis TM, Dyrting KC, Wong CW, Chadwick B, Chan C, Chiang M, Analysis of H5N1 avian influenza infections from wild bird surveillance in Hong Kong from January 2006 to October 2007. Avian Pathol. In press. 10.1080/03079450902751855 PMC4070294 19322709

[18] Smith GJD, Fan XH, Wang J, Li KS, Qin K, Zhang JX, Emergence and predominance of an H5N1 influenza variant in China. Proc Natl Acad Sci U S A. 2006;103:16936–41. 10.1073/pnas.0608157103 17075062 PMC1636557

[19] Guan Y, Poon LLM, Cheung CY, Ellis TM, Lim W, Lipatov AS, H5N1 influenza: a protean pandemic threat. Proc Natl Acad Sci U S A. 2004;101:8156–61. 10.1073/pnas.0402443101 15148370 PMC419573

[20] Viney C, Phillipps K, Ying LC. Birds of Hong Kong and South China, 7th ed. Hong Kong: Government Printer; 1996.

[21] World Health Organization. Towards a unified nomenclature system for the highly pathogenic H5N1 avian influenza viruses. 2007 [cited 2008 May 21]. Available from http://www.who.int/csr/disease/avian_influenza/guidelines/nomenclature/en

[22] Duan L, Bahl J, Smith GJ, Wang J, Vijaykrishna D, Zhang LJ, The development and genetic diversity of H5N1 influenza virus in China, 1996–2006. Virology. 2008;380:243–54. 10.1016/j.virol.2008.07.038 18774155 PMC2651962

[23] Spackman E, Senne DA, Myers TJ, Bulaga LL, Garber LP, Perdue ML, Development of a real-time reverse transcriptase PCR assay for type A influenza virus and the avian H5 and H7 hemagglutinin subtypes. J Clin Microbiol. 2002;40:3256–60. 10.1128/JCM.40.9.3256-3260.2002 12202562 PMC130722

[24] Guan Y, Shortridge KF, Krauss S, Chin PS, Dyrting KC, Ellis TM, H9N2 influenza viruses possessing H5N1-like internal genomes continue to circulate in poultry in southeastern China. J Virol. 2000;74:9372–80. 10.1128/JVI.74.20.9372-9380.2000 11000205 PMC112365

[25] Bao Y, Bolotov P, Dernovoy D, Kiryutin B, Zaslavsky L, Tatusova T, The influenza virus resource at the National Center for Biotechnology Information. J Virol. 2008;82:596–601. 10.1128/JVI.02005-07 17942553 PMC2224563

[26] Nylander JAA. MrModelTest version 2. Evolutionary Biology Centre, Uppsala University, Uppsala, Sweden. 2004 [cited 2008 May 21] Available from http://www.abc.se/~nylander

[27] Swofford DL. PAUP*: phylogenetic analysis using parsimony (and other methods) 4.0 beta. Sunderland (MA): Sinauer Associates; 2001.

[28] Huelsenbeck JP, Ronquist FR. MrBayes: Bayesian inference of phylogenetic trees. Bioinformatics. 2001;17:754–5. 10.1093/bioinformatics/17.8.754 11524383

[29] Ha Y, Stevens DJ, Skehel JJ, Wiley DC. X-ray structures of H5 avian and H9 swine influenza virus hemagglutinins bound to avian and human receptor analogs. Proc Natl Acad Sci U S A. 2001;98:11181–6. 10.1073/pnas.201401198 11562490 PMC58807

[30] Stevens J, Blixt O, Tumpey TM, Taubenberger JK, Paulson JC, Wilson IA. Structure and receptor specificity of the hemagglutinin from an H5N1 influenza virus. Science. 2006;312:404–10. 10.1126/science.1124513 16543414

[31] Yamada S, Suzuki Y, Suzuki T, Le MQ, Nidom CA, Tagawa YS, Hemagglutinin mutations responsible for the binding of H5N1 influenza A viruses to human type receptors. Nature. 2006;444:378–82. 10.1038/nature05264 17108965

[32] Wang J, Vijaykrishna D, Duan L, Bahl J, Zhang JX. Identification of the progenitors of Indonesia and Vietnam avian influenza A (H5N1) viruses from southern China. J Virol. 2008;82:3405–14. 10.1128/JVI.02468-07 18216109 PMC2268469

[33] Treanor JJ, Hayden FG, Vrooman PS, Barbarash R, Bettis R, Riff D, Efficacy and safety of the oral neuraminidase inhibitor oseltamivir in treating acute influenza: a randomized controlled trial. JAMA. 2000;283:1016–24. 10.1001/jama.283.8.1016 10697061

[34] Cheung CL, Rayner JM, Smith GJD, Wang P, Naipospos TSP, Zhang J, Distribution of amantadine-resistant H5N1 avian influenza variants in Asia. J Infect Dis. 2006;193:1626–9. 10.1086/504723 16703504

[35] Roberton SI, Bell DJ, Smith GJ, Nicholls JM, Chan KH, Nguyen DT, Avian Influenza H5N1 in viverrids: implications for wildlife health and conservation. Proc Biol Sci. 2006;273:1729–32. 10.1098/rspb.2006.3549 16790404 PMC1634780

[36] Dung Nguyen T, Vinh Nguyen T, Vijaykrishna D, Webster RG, Guan Y, Malik PJS, Multiple sublineages of influenza A virus (H5N1), Vietnam, 2005–2007. Emerg Infect Dis. 2008;14:632–6. 10.3201/eid1404.071343 18394281 PMC2570938

[37] Starick E, Beer M, Hoffmann B, Staubach C, Werner O, Globig A, Phylogenetic analyses of highly pathogenic avian influenza virus isolates from Germany in 2006 and 2007 suggest at least three separate introductions of H5N1 virus. Vet Microbiol. 2008;128:243–52. 10.1016/j.vetmic.2007.10.012 18031958

[38] World Health Organization. Antigenic and genetic characteristics of H5N1 viruses and candidate H5N1 vaccine viruses developed for potential use as human vaccines. 2008 [cited 2008 May 21]. Available from http://www.who.int/csr/disease/avian_influenza/guidelines/h5n1virus/en/index.html

