# Supplementary material for: Characterization of Avian Influenza Viruses A (H5N1) from Wild Birds, Hong Kong, 2004–2008
Source: Emerg Infect Dis. 2009 Mar;15(3):402–7. doi: 10.3201/eid1503.081190 (PMC2666293; doi:10.3201/eid1503.081190)
Supplement: Appendix Table — Influenza viruses A (H5N1) isolated in Hong Kong, China, 2004-2008 [file 08-1190_appT-s1.pdf]

Appendix Table. Influenza viruses A (H5N1) isolated in Hong Kong, China, 2004–2008

Virus	Submission date	Host scientific name*	Clade†	Genotype‡	Reference
Peregrine falcon/HK/D0028/2004	2004 Jan 21	*Falco peregrinus*	9	Z	(*1*)
Grey heron/HK/728/2004	2004 Nov 1	*Ardea cinerea*	2.3.2	V2	(*7*)
Grey heron/HK/837/2004	2004 Dec 8	*Ardea cinerea*	2.3.2	V2	(*7*)
Chinese pond heron/HK/18/2005	2005 Jan 10	*Ardeola bacchus*	2.3.2	V2	(*7*)
Magpie robin/HK/75/2006	2006 Jan 10	*Copsychus saularis*	2.3.4	V	(*18*)
Magpie robin/HK/366/2006	2006 Jan 26	*Copsychus saularis*	2.3.4	V	(*18*)
Crested myna/HK/540/2006	2006 Jan 31	*Acridotheres cristatellus*	2.3.4	V	(*18*)
Chicken/HK/282/2006	2006 Jan 31	*Gallus gallus*	2.3.4	V	(*18*)
Common magpie/HK/645/2006	2006 Feb 3	*Pica pica*	2.3.4	V	(*18*)
Little egret/HK/718/2006	2006 Feb 3	*Egretta garzetta*	2.3.4	V	(*18*)
Chicken/HK/947/2006	2006 Feb 6	*Gallus gallus*	2.3.4	V	(*18*)
Japanese white-eye/HK/1038/2006	2006 Feb 7	*Zosterops japonica*	2.3.4	G	(*18*)
Common magpie/HK/2125/2006	2006 Feb 16	*Pica pica*	2.3.4	G	(*18*)
Common magpie/HK/2256/2006	2006 Feb 17	*Pica pica*	2.3.4	G	(*18*)
Munia/HK/2454/2006	2006 Feb 20	*Lonchura sp.*	2.3.4	G	(*18*)
White-backed munia/HK/2469/2006	2006 Feb 20	*Lonchura striata*	2.3.4	G	(*18*)
Large-billed crow/HK/2512/2006	2006 Feb 20	*Corvus macrorhynchus*	2.3.4	G	(*18*)
House crow/HK/2648/2006	2006 Feb 21	*Corvus splendens*	2.3.4	G	(*18*)
House crow/HK/2858/2006	2006 Feb 23	*Corvus splendens*	2.3.4	G	(*18*)
Common magpie/HK/3033/2006	2006 Feb 25	*Pica pica*	2.3.4	G	(*18*)
Peregrine falcon/HK/5211/2006	2006 Mar 22	*Falco peregrinus*	2.3.2	G	–§
Scaly-breasted munia/HK/45/2007	2007 Jan 3	*Lonchura punctulata*	2.3.4	V1	–
Crested goshawk/HK/458/2007	2007 Jan 12	*Accipiter trivirgatus*	2.3.4	V1	–
House crow/HK/719/2007	2007 Jan 17	*Corvus splendens*	2.3.4	V	–
Japanese white-eye/737/2007	2007 Jan 17	*Zosterops japonica*	2.3.4	V	–
White-backed munia/HK/828/2007	2007 Jan 18	*Lonchura striata*	2.3.4	V	–
Peregrine falcon/HK/1143/2007	2007 Jan 24	*Falco peregrinus*	2.3.2	V	–
House crow/HK/1203/2007	2007 Jan 25	*Corvus splendens*	2.3.4	V	–
Blue magpie/HK/1993/2007	2007 Feb 7	*Urocissa caerulea*	2.3.4	V	–
Silver-eared mesia/2065/2007	2007 Feb 9	*Leiothrix argentauris*	2.3.4	V	–
Common kestrel/HK/2372/2007	2007 Feb 17	*Falco tinnunculus*	2.3.4	V	–
Scaly-breasted munia/2433/2007	2007 Feb 21	*Lonchura punctulata*	2.3.4	V	–
Chestnut munia/HK/2442/2007	2007 Feb 21	*Lonchura atricapilla*	2.3.4	V	–
Scaly-breasted munia/HK/2572/2007	2007 Feb 27	*Lonchura punctulata*	2.3.4	V	–
Long-tailed shrike/HK/2762/2007	2007 Mar 6	*Lanius schach*	2.3.4	V1	–
Red-billed starling/HK/4925/2007	2007 May 28	*Sturnus sericeus*	2.3.2	V	–
Common magpie/HK/5052/2007	2007 Jun 4	*Pica pica*	2.3.2	V	–
Daurian starling/HK/1532/2007	2007 Jun 5	*Sturnus sturninus*	2.3.2	V	–
House crow/HK/5288/2007	2007 Jun 12	*Corvus splendens*	2.3.2	V	–
Little egret/HK/8550/2007	2007 Nov 20	*Egretta garzetta*	2.3.2	V (novel variant)	–
Little egret/HK/8863/2007	2007 Dec 5	*Egretta garzetta*	2.3.2	V	–
Grey heron/HK/3088/2007	2007 Dec 6	*Ardea cinerea*	2.3.2	V	–
Common buzzard/HK/9213/2007	2007 Dec 18	*Buteo buteo*	2.3.2	V	–
Black-crowned night heron/HK/659/2008	2008 Jan 29	*Nycticorax nycticorax*	2.3.2	V	–
Great egret/HK/807/2008	2008 Feb 4	*Ardea alba*	2.3.2	V	–
Grey heron/HK/1046/2008	2008 Feb 9	*Ardea cinerea*	2.3.2	V	–
Magpie robin/HK/1097/2008	2008 Feb 11	*Copsychus saularis*	2.3.2	V	–
Magpie robin/HK/1897/2008	2008 Mar 3	*Copsychus saularis*	2.3.2	V	–
Peregrine falcon/HK/2142/2008	2008 Mar 10	*Falco peregrinus*	2.3.4	V (novel variant)	–

*Scientific and common names from Viney et al. (*20*).
†Clade designations according to the World Health Organization influenza (H5N1) nomenclature system (*21*).
‡Genotypes according to Duan et al. (*22*).
§Characterized in this study.
